# Evaluation of Parasite Concentrator Kit and Kato–Katz Method for Detection of Intestinal Parasites in Stool Samples

**DOI:** 10.3390/tropicalmed11050118

**Published:** 2026-04-29

**Authors:** Penchom Janwan, Lakkhana Sadaow, Patcharaporn Boonroumkaew, Rutchanee Rodpai, Oranuch Sanpool, Tongjit Thanchomnang, Pokkamol Laoraksawong, Pewpan Maleewong Intapan, Wanchai Maleewong

**Affiliations:** 1 Department of Medical Technology, School of Allied Health Sciences, Walailak University, Nakhon Si Thammarat 80160, Thailand; penchom.ja@wu.ac.th; 2 Hematology and Transfusion Science Research Center, Walailak University, Nakhon Si Thammarat 80160, Thailand; 3 Mekong Health Science Research Institute, Khon Kaen University, Khon Kaen 40002, Thailand; lakkhasa@kku.ac.th (L.S.); paboon@kku.ac.th (P.B.); rutcro@kku.ac.th (R.R.); tongjit.t@msu.ac.th (T.T.); pewpan@kku.ac.th (P.M.I.); wanch_ma@kku.ac.th (W.M.); 4 Department of Parasitology, Faculty of Medicine, Khon Kaen University, Khon Kaen 40002, Thailand; 5 Faculty of Medicine, Mahasarakham University, Maha Sarakham 44000, Thailand; 6 Department of Occupational Safety and Environmental Health, Faculty of Public Health, Khon Kaen University, Khon Kaen 40002, Thailand; pokkla@kku.ac.th

**Keywords:** helminthiases, Thailand, fecal parasite concentrator kit, Kato–Katz, intestinal parasites, diagnostic accuracy

## Abstract

Background: To address the significant burden of helminthiases in Thailand, this cross-sectional study compared the performance of a fecal parasite concentrator kit (FPCK) against the Kato–Katz (KK) method for diagnosing intestinal parasites in endemic populations across the Northeast and Southern regions. Methods: Stool samples were collected from 140 participants and examined for intestinal parasitic infections using both FPCK and KK methods. Results: The FPCK method demonstrated a significantly higher detection rate of 45.0% compared to 35.0% for the KK method. For detecting liver fluke (*Opisthorchis viverrini*), the FPCK method detected significantly more cases than the KK method (10.71% vs. 4.29%) (*p* = 0.0027). For other parasites such as *Trichuris trichiura*, *Strongyloides stercoralis*, and *Entamoeba coli*, the FPCK method tended to detect more infections, but the differences were not statistically significant (*p* > 0.05). Conclusions: The FPCK method showed better performance than the KK method for detecting intestinal helminth infections in stool samples, particularly *O. viverrini*, *T. trichiura*, *S. stercoralis*, and *Entamoeba coli*. Therefore, FPCK could be used as a suitable stool examination method for surveillance and monitoring of preventive treatment for opisthorchiasis.

## 1. Introduction

Helminthiases, a major group of neglected tropical diseases (NTDs), are parasitic infections caused by a diverse array of parasitic worms that are predominantly found in tropical and subtropical regions [1,2]. Opisthorchiasis, a foodborne helminth (FBH) infection is a major public health problem in five countries of the Greater Mekong Subregion: Thailand, Laos, Myanmar, Cambodia, and Vietnam [3]. It is estimated that 12.39 million people are affected by *Opisthorchis viverrini*, with the highest burden concentrated in the Lower Mekong Basin [4]. In Thailand, *O. viverrini* represents a particularly serious public health concern, especially in the northeastern region, where at least 6 million people are estimated to be infected [5,6]. *Opisthorchis viverrini* infection occurs following the consumption of raw cyprinid fish dishes containing metacercariae. Upon ingestion, the motile juvenile fluke excysts and migrates into the common bile duct through the ampulla of Vater, subsequently maturing into the adult form of the liver fluke within the intrahepatic bile ducts. These parasites inhabit the biliary tract for several years, producing eggs that pass into the bile and are ultimately excreted from the host through the feces [7]. Controlling opisthorchiasis is particularly challenging due to the parasite’s complex life cycle, which involves multiple hosts, diverse environmental conditions, and an intricate disease transmission process [7]. Chronic *O. viverrini* infection is strongly associated with hepatobiliary diseases and cholangiocarcinoma, a primary form of liver cancer prevalent in this region [8]. The combined costs of medical care and loss of productivity resulting from *O. viverrini* infection in Thailand are estimated at approximately USD $120 million annually [9].

Additionally, soil-transmitted helminth (STH) infection by *Trichuris trichiura* affects approximately 464 million people globally, leading to a significant burden of 0.64 million disability-adjusted life years (DALYs) lost annually [10,11]. Moreover, *T. trichiura* infection persists as an important public health problem in Asian regions [12]. In Thailand, *T. trichiura* remains a prevalent health issue, with the highest infection rates consistently reported in the southern region due to its favorable environmental conditions for parasite transmission [13]. Infection often presents subclinically or in a mild form; however, severe infections, particularly in children, can result in weight loss, malnutrition, anemia, and rectal prolapse, in addition to watery stools, mucoid discharge, abdominal pain, nausea, and vomiting [14].

Human strongyloidiasis is also a significant intestinal helminthic disease, affecting approximately 300 to 600 million people worldwide [15,16]. In 2020, more than 2.6 billion people were estimated to be at risk of *Strongyloides* infection globally, with the greatest burden of disease disproportionately affecting individuals in low- and middle-income countries [17]. An approximate prevalence of 15.7% has been reported in the northeastern region of Thailand [18]. Asymptomatic carriers with chronic strongyloidiasis may develop hyperinfection syndrome if they become immunocompromised, and fatal disseminated strongyloidiasis can occur in immunosuppressed patients, specifically, those receiving systemic corticosteroids or cytotoxic treatments, such as antineoplastic agents [19].

Prompt diagnosis is critically important for the treatment and public health control of FBH and STH infections. Detection of parasitic objects in stool samples is considered the ‘gold-standard’ diagnostic method for opisthorchiasis [20], trichuriasis [21], and strongyloidiasis [16]. However, since direct microscopic examination of stool samples has low sensitivity for detecting parasitic agents, various concentration techniques have been introduced to enhance the sensitivity of microscopic detection. These techniques separate parasites from fecal debris and improve clarity, enabling the detection of parasitic agents even at low parasite intensities, such as the formalin–ethyl acetate sedimentation concentration technique [22] and the Kato–Katz (KK) method [23]. Here, we present a comparative study of a commercially available fecal parasite concentrator kit (FPCK) and the KK method for detecting intestinal parasites using human stool samples from the northeast (opisthorchiasis and strongyloidiasis-endemic area) and southern (trichuriasis-endemic area) regions of Thailand. This study may provide guidance on the method of choice for stool examination of these intestinal helminths in specimens with low parasite intensity.

## 2. Materials and Methods

### 2.1. Study Area and Population

Stool specimens were collected during fieldwork in 2020 from ten districts (Chiang Yuen, Kantharawichai, Na Dun, Kae Dam, Phayakkhaphum Phisai, Yang Si Surach, Wapi Pathum, Na Chueak, Kut Rang, and Borabue) in Maha Sarakham Province, northeast Thailand, and in 2024 from Tha Sala District in Nakhon Si Thammarat Province, southern Thailand. The sample size was determined using the sample size estimation formula for diagnostic tests (Hajian-Tilaki, 2014) [24]. It was calculated using a prevalence rate (p) of 32.0%, as detailed in the Kopolrat et al. (2022) [25] study, with a 95% confidence interval (z = 1.96) and a 5% margin of error (d = 0.05). The calculated sample size was 178 people. A total of 140 subjects (78.65%) returned stool specimens.

### 2.2. Field Procedure and Sample Collection

A single stool specimen was processed using two microscopy-based examination methods: the KK method [23] and FPCK (J. SLIP Engineering CO. LTD., Samut Sakhon, Thailand) to identify parasitic organisms. The Kato–Katz technique was performed in the field within one day of specimen collection. Remaining stool samples were stored at 4 °C and processed within two weeks using FPCK at the Department of Parasitology, Faculty of Medicine, Khon Kaen University. The specimens were blinded using a code-embedded procedure.

### 2.3. Detection by Kato–Katz (KK) Method

One gram of feces was pressed through a mesh screen to remove large particles. A portion of the sieved sample was then transferred into the hole of a template positioned on a slide. After filling the hole completely, the template was removed, and the remaining sample (yielding a final mass of 0.038 g) was covered with a piece of cellophane that had been pre-soaked in a glycerol-malachite green solution. The parasite eggs were counted under a light microscope [23].

### 2.4. Detection by Fecal Parasite Concentrator Kit (FPCK)

The remaining stool sample from KK method was scooped using the spoon (yielding a final mass of 0.50 g) attached to the end of the FPCK (J. SLIP Engineering). The fecal specimens were homogenized with the provided solvent containing preservative reagents vortexed to achieve emulsification and subsequently passed through an incorporated mesh filter (pore size of approximately 250 µm), with the sedimentation cone positioned upward. The tube was then inverted and centrifuged at 500 g for 2 minutes, after which the top layers of the supernatant were decanted. Four drops of 0.85% saline were added to the sediment, and the resulting suspension was examined under a light microscope.

### 2.5. Statistical Analysis

The results from both methods were compared using the Chi-square test, with *p* < 0.05 considered statistically significant. McNemar’s test was used to evaluate differences in paired parasite detection proportions. Sensitivity, specificity, positive predictive value, and negative predictive value were calculated. Interrater agreement was assessed using Cohen’s kappa test.

## 3. Results

A total of 140 participants were enrolled in the study, comprising 80 males (57.14%) and 60 females (42.86%). The most common age group was ≤30 years, accounting for 43.8% of the 137 participants with complete age information (median age: 43 years; range: 6–79 years) (Table 1). 

When comparing the detection rates of intestinal parasites using the KK and FPCK methods, the FPCK method demonstrated a significantly higher detection rate of 45.0% compared to 35.0% for the KK method (Table 2). Using both methods, detection rates were higher in males than in females (FPCK: 26.43% vs. 18.57%; KK: 22.14% vs. 12.86%), and the ≤30 years age group exhibited the highest infection rates (Table 2). 

Regarding parasite-specific detection, a statistically significant difference (*p* = 0.0027) was observed between the KK and FPCK methods for detecting liver fluke (*Opisthorchis viverrini*), with the FPCK method detecting significantly more cases than the KK method (10.71% vs. 4.29%). For other parasites such as *Trichuris trichiura*, *Strongyloides stercoralis*, and *Entamoeba coli*, the FPCK method tended to detect more infections, but the differences were not statistically significant (*p* > 0.05) (Table 3). Quantitative data on infection intensity (eggs per gram) are presented in Supplementary Table S1. Comparative analysis indicates that the KK method yields higher estimated egg burdens than the FPCK method, whereas the FPCK method demonstrates superior detection of protozoa.

The diagnostic accuracy of the FPCK method was comparatively evaluated against the KK method. The FPCK method showed a sensitivity of 69.8%, specificity of 93.5%, positive predictive value of 89.8%, and negative predictive value of 79.1% (Table 4). The Kappa coefficient, used to measure inter-method agreement using Cohen’s kappa, was 64.7% (95% CI: 52.1–77.2,), indicating moderate to good correlation and statistically significant agreement (*p* < 0.0001).

## 4. Discussion

We evaluated the performance of FPCK and KK for the diagnosis of intestinal parasitic infections in Thailand, the endemic area of opisthorchiasis, trichuriasis and strongyloidiasis [6,10,18]. From the present study, FPCK method showed a higher sensitivity for diagnosing intestinal helminth infections, particularly for *O. viverrini*, *T. trichiura*, *S. stercoralis*, and *E. coli* than the KK method. However, for quantitative egg burden assessment, the KK method yields higher values, likely because it utilizes unprocessed stool without partial removal of material. The superior parasite diversity detected by the FPCK method may be attributed to several methodological advantages over the KK technique. These include the use of a comparatively larger stool volume, an integrated filtration step, and the application of a fat-separation solution, collectively yielding a cleaner sediment and thereby enhancing diagnostic sensitivity. The KK method, by contrast, is subject to inherent limitations: a reduced quantity of stool examined, the limited of procedural time constraints, and the potential for constituent reagents to alter the morphology of helminth eggs or larvae. Furthermore, the KK technique is not suitable for the detection of protozoan infections, restricting its applicability in settings where mixed or protozoan parasitic infections are prevalent. With respect to *Strongyloides stercoralis*, interpretation of the comparative performance of the FPCK method warrants particular caution. The KK technique is not generally regarded as an optimal reference method for strongyloidiasis, given its reliance on the detection of clearing larvae that are readily distorted by glycerol and are present only transiently and in low numbers in stool specimens. Consequently, any apparent superiority of the FPCK method in detecting *S. stercoralis* may, in part, reflect the inherent insensitivity of the KK method as a comparator rather than a true measure of FPCK diagnostic performance.

Moreover, a potential source of bias in this study is the difference in sample handling between methods. KK was performed within one day of stool collection, whereas FPCK samples were stored at 4 °C and processed up to two weeks later. This delay may affect parasite integrity or egg recovery, potentially influencing detection rates. Although refrigeration may help preserve some parasite stages, differential effects between methods cannot be excluded and may have contributed to the observed differences in diagnostic performance. A potential limitation of this study is the possibility of obtaining a smaller sample than the calculated size, which may compromise statistical power and reduce the reliability of findings.

This finding is supported by previous studies [26,27]. Previously, the FPCK method using Mini Parasep® SF stool parasite concentrator technique performs better than the KK thick smear for diagnosing intestinal helminth infections, particularly *Schistosoma mansoni*, *Ascaris lumbricoides* and *Hymenolepis nana* infections in stool samples, while the KK technique is better for the diagnosis of *T. trichiura* [26]. However, Charoensuk et al. 2019 [28] reported that the formalin–ethyl acetate sedimentation concentration technique showed the highest efficiency to detect *O. viverrini* egg in stool, followed by Kato–Katz technique, FPCK and direct simple smear technique. The discrepancies in FPCK performance across various studies may be attributed not only to the brand of the kit and the expertise of microscopists but also to variations in technical protocols. Factors such as centrifugation speed, the duration of the sedimentation process, and the efficiency of the filtration system (mesh size) play a critical role in the recovery rate of small parasite eggs like *O. viverrini*. Nevertheless, a significant advantage of FPCK technique was the decreased turnaround time and faster reporting than the conventional formal ethyl acetate sedimentation technique [27]. In addition, FPCK employs a safety lock container, so the disposable enclosed system can prevent the release of chemicals and contamination and constitutes a less hazardous procedure, compared with the conventional formal ethyl acetate sedimentation technique. Furthermore, a critical limitation of the Kato–Katz method is its time-dependency; slides must be examined within 30 to 60 minutes of preparation to prevent the over-clearing of thin-shelled helminth eggs, such as hookworms, which can lead to false-negative results. In contrast, the FPCK technique utilizes a fixative-based system that preserves the morphology of parasite eggs, larvae, and protozoan cysts for extended periods. This stabilization approach obviates the necessity for immediate microscopic examination, thereby conferring greater flexibility for future large-scale epidemiological screenings and ensuring markedly enhanced diagnostic reliability across resource-limited or high-volume laboratory settings. Furthermore, the inherent cost-effectiveness of the FPCK method—characterized by its relatively low reagent requirements, minimal infrastructure dependencies, and reduced need for highly specialized technical personnel—presents a highly promising prospect for economic feasibility within large-scale parasitic control programs. Such attributes collectively suggest that FPCK holds considerable potential for sustainable integration into national and international public health strategies, particularly in endemic regions where programmatic scalability and financial viability remain paramount considerations for long-term disease control and elimination efforts.

## 5. Conclusions

In conclusion, comparative evaluation of the FPCK method versus the KK method demonstrated acceptable sensitivity, specificity, positive and negative predictive values, with moderate to good inter-method agreement. These results suggest that the FPCK method (which operates on a similar principle to the Mini Parasep® SF technique) holds promise as a complementary or alternative diagnostic tool to the KK method for the detection of intestinal parasites in selected surveillance contexts, rather than a wholesale replacement. Notably, the FPCK method demonstrated effective detection of intestinal protozoan cysts, a capacity absent in the KK technique. The FPCK may also serve as a suitable fecal examination method for the surveillance and monitoring of preventive chemotherapy programs targeting helminthiasis, particularly in areas endemic for opisthorchiasis—including infections with *Opisthorchis viverrini*—as well as trichuriasis, and strongyloidiasis. Nevertheless, the selection of an appropriate diagnostic method should ultimately be guided by the predominant parasite species within the target community and the specific objectives of the study.

## Figures and Tables

**Table 1:** Baseline characteristics of study samples.

Characters	Number	Percentage
Gender		
Male	80	57.14
Female	60	42.86
Age (N = 137)		
≤30	60	43.8
31–40	4	2.92
41–50	16	11.68
51–60	31	22.63
>60	26	18.98
Median (min: max)	43 (6: 79)	

**Table 2:** Prevalence of intestinal parasites examined by Kato–Katz (KK) and Fecal parasite concentrator kit (FPCK) techniques.

Characters	Examination Methods			
	KK		FPCK	
	No. Positive	Percentage (95% CI)	No. Positive	Percentage (95% CI)
Total	49	35.00 (27.14–43.51)	63	45.00 (36.59–53.63)
Gender				
Male	31	22.14 (15.57–29.93)	37	26.43 (19.34–35.54)
Female	18	12.86 (7.80–19.56)	26	18.57 (12.50–26.01)
Age (N = 137)				
≤30	28	20.43 (14.03–28.16)	30	21.9 (15.29–29.76)
31–40	2	1.46 (0.18–5.17)	2	1.46 (0.18–5.17)
41–50	3	2.19 (0.45–6.27)	7	5.11 (2.08–10.24)
51–60	8	5.84 (2.55–11.18)	10	7.3 (3.56–13.01)
>60	8	5.84 (2.55–11.18)	13	9.49 (5.15–15.68)

**Table 3:** Prevalence of intestinal parasites by Kato–Katz (KK) and fecal parasite concentrator kit (FPCK) techniques and statistical comparisons between techniques.

Parasites	No. Positive (%)		*p*-Value
	KK	FPCK	
*Opisthorchis viverrini*	6 (4.29)	15 (10.71)	0.0027 *
*Trichuris trichiura*	28 (20.0)	30 (21.43)	0.1573
*Strongyloides stercoralis*	4 (2.86)	7 (5.00)	0.0833
*Taenia* spp.	1 (0.71)	2 (1.43)	0.3173
Echinostome egg	1 (0.71)	1(0.71)	omitted
MIF	0 (0)	2 (1.43)	0.1573
Hookworm	0 (0)	1(0.71)	0.3173
*Sarcocystis* spp.	1 (0.71)	2 (1.43)	0.5637
*Entamoeba coli*	2 (1.43)	5 (3.57)	0.0833
*Blastocystis hominis*	0 (0)	2 (1.43)	0.1573
*Giardia lamblia*	0 (0)	1(0.71)	0.3173

* *p*-value < 0.05 and compare by McNemar’s test. The number exceeded the total positive count due to duplicate counting of parasite species in cases with multiple co-infections.

**Table 4:** Diagnostic accuracy and agreements of FPCK vs KK in the diagnostic intestinal parasites.

Accuracy	Value	95% CI
Sensitivity	69.80%	57.0–80.80
Specificity	93.50%	85.5–97.90
Positive predictive value	89.80%	77.80–96.60
Negative predictive value	79.10%	69.30–86.90
Agreements Kappa	64.70%	52.10–77.20 **

** *p*-value < 0.0001. Inter-method agreement was evaluated using Cohen’s kappa, with statistically significant agreement observed.

## Data Availability

All data and tables presented in this manuscript are original.

## Supplementary Materials

The following supporting information can be downloaded at: https://www.mdpi.com/article/10.3390/tropicalmed11050118/s1, Table S1: Quantitative results of microscopic examination for parasitic infections using the Kato–Katz (KK) and Fecal Parasite Concentrator Kit (FPCK) techniques.

## Abbreviations

The following abbreviations are used in this manuscript:

FPCK	Fecal Parasite Concentrator Kit
KK	Kato–Katz
NTDs	Neglected Tropical Diseases
FBH	Foodborne Helminth
