# Supplementary material for: Evaluation of Parasite Concentrator Kit and Kato–Katz Method for Detection of Intestinal Parasites in Stool Samples
Source: Trop Med Infect Dis. 2026 Apr 29;11(5):118. doi: 10.3390/tropicalmed11050118 (PMC13211567; doi:10.3390/tropicalmed11050118)
Supplement: Supplementary file 1 [file tropicalmed-11-00118-s001.zip › tropicalmed-4229111-supplementary.pdf]

Supplementary Table S1. Quantitative results of microscopic examination for parasitic infections using the Kato-Katz (KK) and Fecal Parasite Concentrator Kit (FPCK) techniques.

No.	Kato	EPG	JK	EPG
1	Not found	Not done	Not found	0
2	Not found	Not done	*Opisthorchis viverrini*	13
3	*O. viverrini*	23	Not found	0
4	Not found	0	Not found	0
5	*Taenia* spp.	Not done	*Taenia* spp.	Not done
6	Not found	Not done	Not found	Not done
7	Not found	Not done	Not found	0
8	Not found	Not done	Hookworm	19
9	Not found	Not done	Not found	0
10	Not found	Not done	Not found	0
11	Not found	Not done	Not found	0
12	*O. viverrini*	23	*O. viverrini*	27
	Strogyloides stercoralis	23	Not found	0
13	*O. viverrini*	138	Not found	0
	S. stercoralis	46	Not found	0
14	Not found	Not done	Not found	0
15	*O. viverrini*	575	*O. viverrini*	39
16	Not found	Not done	Not found	0
17	*O.viverrini*	184	*O. viverrini*	35
	Taenia spp.	Not done	*Taenia* spp.	Not done
	S. stercoralis	23	Not found	0
18	*O. viverrini*	46	Not found	0
	Echinostome	345	Echinostome	68
19	Not found	Not done	Minute intestinal fluke	15
20	Not found	Not done	Not found	0
21	Taenia spp.	115	Not found	0
22	Not found	0	*En. coli*	0
23	*O. viverrini*	667	*O. viverrini*	39
24	*O. viverrini*	4117	*O. viverrini*	160
			Minute intestinal fluke	12
25	Not found	Not done	*O. viverrini*	12
26	Not found	Not done	Not found	0
27	Not found	Not done	Not found	0
28	*O. viverrini*	138	*O. viverrini*	13
29	Not found	Not done	Not found	0
30	Not found	Not done	Not found	0
31	Not found	Not done	Not found	0
32	Not found	Not done	Not found	0
33	Not found	Not done	*O. viverrini*	112
34	Not found	Not done	Not found	0
35	*O. viverrini*	138	Not found	0
			En. coli	0
36	Not found	Not done	*S. stercoralis*	39
37	Not found	Not done	Not found	0
38	Not found	Not done	Not found	0

39	Not found	Not done	*S. stercoralis*	54
			Sarcocystis spp.	
40	Not found	Not done	Not found	0
41	Not found	Not done	*S. stercoralis*	32
42	Not found	Not done	Not found	0
43	Not found	Not done	Not found	0
44	Not found	Not done	*S. stercoralis*	156
			Sarcocystis spp.	
45	Not found	Not done	Not found	0
46	Not found	Not done	Not found	0
47	*O.viverrini*	23	*O.viverrini*	17
48	Not found	Not done	*Blastocystis* spp.	0
49	Not found	Not done	Not found	0
50	Not found	Not done	*Sarcocystis* spp.	0
51	Not found	Not done	Not found	0
52	*O.viverrini*	23	Not found	0
53	Not found	Not done	*S. stercoralis*	17
54	Not found	Not done	Not found	0
55	Not found	Not done	Not found	0
56	Not found	Not done	Not found	0
57	Not found	Not done	Not found	0
58	Not found	Not done	*O. viverrini*	13
59	Not found	Not done	Not found	0
60	*S. stercoralis*	46	*S. stercoralis*	42
61	Not found	Not done	Not found	0
62	Not found	Not done	Not found	0
63	Not found	Not done	*O. viverrini*	16
64	Not found	Not done	Not found	0
65	*O. viverrini*	322	*O. viverrini*	120
66	Not found	Not done	Not found	0
67	Not found	Not done	Not found	0
68	Not found	Not done	Not found	0
69	Not found	Not done	Not found	0
70	Not found	Not done	*En. coli*	0
71	*O. viverrini*	23	Not found	0
			En. coli	0
			Blastocystis spp.	
72	Not found	Not done	Not found	0
73	Not found	Not done	*S. stercoralis*	60
74	Not found	Not done	Not found	0
75	Not found	Not done	Not found	0
76	Not found	Not done	Not found	0
77	*O. viverrini*	46	Not found	0
78	Echinostome	115	Echinostome	112
79	*O. viverrini*	92	*O.viverrini*	89
80	*O. viverrini*	138	*O.viverrini*	34
81	*Trichuris trichiura*	23	*T. trichiura*	15
82	*T. trichiura*	732	*T. trichiura*	23
83	*T. trichiura*	137	*T. trichiura*	14

84	*T. trichiura*	46	*T. trichiura*	10
85	*T. trichiura*	23	*T. trichiura*	13
86	*T. trichiura*	46	*T. trichiura*	35
			G. lamblia	ND
87	*T. trichiura*	23	*T. trichiura*	13
88	*T. trichiura*	69	*T. trichiura*	35
			En. coli	ND
89	*T. trichiura*	435	*T. trichiura*	163
90	*T. trichiura*	46	*T. trichiura*	4
91	*T. trichiura*	46	*T. trichiura*	11
92	*T. trichiura*	46	*T. trichiura*	47
93	*T. trichiura*	160	*T. trichiura*	80
94	*T. trichiura*	595	*T. trichiura*	231
95	*T. trichiura*	92	*T.trichiura*	56
96	*T. trichiura*	275	*T. trichiura*	20
97	*T. trichiura*	2426	*T. trichiura*	217
98	*T. trichiura*	114	*T. trichiura*	26
99	*T. trichiura*	46	*T. trichiura*	36
100	*T. trichiura*	69	*T. trichiura*	114
101	*T. trichiura*	160	*T. trichiura*	9
102	*T. trichiura*	206	*T. trichiura*	33
103	*T. trichiura*	23	*T. trichiura*	6
104	*T. trichiura*	2334	*T. trichiura*	424
105	*T. trichiura*	23	*T. trichiura*	15
106	*T. trichiura*	69	*T. trichiura*	34
107	*T. trichiura*	46	*T. trichiura*	8
108	*T. trichiura*	549	*T. trichiura*	1233
109	Not found	Not done	Not found	Not done
110	Not found	Not done	Not found	Not done
111	Not found	Not done	Not found	Not done
112	Not found	Not done	Not found	Not done
113	Not found	Not done	Not found	Not done
114	Not found	Not done	Not found	Not done
115	Not found	Not done	Not found	Not done
116	Not found	Not done	Not found	Not done
117	Not found	Not done	Not found	Not done
118	Not found	Not done	Not found	Not done
119	Not found	Not done	Not found	Not done
120	Not found	Not done	Not found	Not done
121	Not found	Not done	Not found	Not done
122	Not found	Not done	Not found	Not done
123	Not found	Not done	Not found	Not done
124	Not found	Not done	Not found	Not done
125	Not found	Not done	Not found	Not done
126	Not found	Not done	Not found	Not done
127	Not found	Not done	Not found	Not done
128	Not found	Not done	Not found	Not done
129	Not found	Not done	Not found	Not done
130	Not found	Not done	Not found	Not done

131	Not found	Not done	Not found	Not done
132	Not found	Not done	Not found	Not done
133	Not found	Not done	Not found	Not done
134	Not found	Not done	Not found	Not done
135	Not found	Not done	Not found	Not done
136	Not found	Not done	Not found	Not done
137	Not found	Not done	Not found	Not done
138	Not found	Not done	Not found	Not done
139	Not found	Not done	Not found	Not done
140	Not found	Not done	Not found	Not done
